# Supplementary material for: The chiropractors’ dilemma in caring for older patients with musculoskeletal complaints: Collaborate, integrate, coexist, or separate?
Source: PLoS One. 2024 May 2;19(5):e0302519. doi: 10.1371/journal.pone.0302519 (PMC11065304; doi:10.1371/journal.pone.0302519)
Supplement: S1 Appendix — (PDF) [file pone.0302519.s001.pdf]

Interview guide

Question areas

Probing questions: what do you mean…, can you give some examples…,

Generally

1. How would you describe the typical 55+ patient that you meet in your clinical practice?

2. In your view, what is the most common complaint that patients 55+ seeking care for?

3. Why do you think patients come to you as a chiropractor for their problems?

4. In your experience, have the help-seeking behaviours of patient 55+ visiting a chiropractor for their MSK complaints changed? What kind of changes have you noticed? Over what period of time have you noticed these changes?

Treatment-related issues and clinical challenges regarding MSK complaints

1. What do you find to be the most challenging part when it comes to treatment and assessment of patients 55+? Can you give some examples?

2. In your experience, what do you feel is the biggest difference in clinical management and care when it comes to patients 55+ compared to younger patients?

3. Could you describe your experiences regarding treatment/care in the case of comorbidity in patients and/or of patients with chronic underlying diseases?

4. What do you think would is necessary to improve the treatment/care of patients 55+?

5. Is there something you believe that were missing in your education when it comes to the care and management of patients 55+? If so, what do you believe were missing?

Collaboration with other professions regarding patients 55+ and MSK complaints

1. What is your experience regarding the referral procedure for patients 55+? Does it matter the direction of the referral - to the chiropractor or from the chiropractor?

2. What is your experience regarding the collaboration with other healthcare professions when it comes to the co-management of patients 55+?

3. How do you perceive the communication between you as a chiropractor and other healthcare professions when it comes to patients 55+ *?*

4. Have you experienced conflicts that may arise regarding the clinical management of a patient between you and other professions in the healthcare system? If so, what was the reason for the conflicts?

Drugs and lifestyle in patients 55+ and MSK complaints

1. On a scale of 0 to 10, where 10 is very good knowledge, how would you rate your pharmacological knowledge?

2. According to your experience in the treatment and management of this patient group, what short- and long-term consequences do you experience that MSK complaints can lead to for the patient?

3. On a scale of 0 to 10, where 10 is very good support and help, how would you rate your ability to provide support and help to the patient?

4. In your experience, what opportunities do you have as a chiropractor to influence the patient's lifestyle? Are there certain lifestyle issues and if so which ones?

The chiropractor's view regarding the future and treatment of the patient group

1. In your view, when it comes to chiropractic treatment/management of patients 55+, what do you see in the future?

2. At present, what shortcomings/strengths do you think there are regarding the treatment/management of patients 55+?

3. If you can think freely, without having to worry about the implementation or costs, what do you think the treatment/management of patients 55+ with MSK would look like?

4. What knowledge would you need to improve the treatment/care of patients 55+?

Other

1. Is there any area that we have not already talked about that you think is important to address regarding the chiropractic management of patients over 55 with MSK disorders?

References used for the interview guide

Beaudart C, Biver E, Bruyere O, Cooper C, Al-Daghri N, Reginster JY, et al. Quality of life assessment in musculo-skeletal health. Aging Clin Exp Res. 2018;30(5):413-8.

Beliveau PJH, Wong JJ, Sutton DA, Simon NB, Bussieres AE, Mior SA, et al. The chiropractic profession: a scoping review of utilization rates, reasons for seeking care, patient profiles, and care provided. Chiropr Man Therap. 2017;25:35.

de Luca K, Wong A, Eklund A, Fernandez M, Byles JE, Parkinson L, et al. Multisite joint pain in older Australian women is associated with poorer psychosocial health and greater medication use. Chiropr Man Therap. 2019;27:8.

Dionne CE, Dunn KM, Croft PR. Does back pain prevalence really decrease with increasing age? A systematic review. Age Ageing. 2006;35(3):229-34.

Fejer R, Ruhe A. What is the prevalence of musculoskeletal problems in the elderly population in developed countries? A systematic critical literature review. Chiropr Man Therap. 2012;20(1):31.

Foster NE, Hartvigsen J, Croft PR. Taking responsibility for the early assessment and treatment of patients with musculoskeletal pain: a review and critical analysis. Arthritis Res Ther. 2012;14(1):205.

Hartvigsen J, Frederiksen H, Christensen K. Back and neck pain in seniors-prevalence and impact. Eur Spine J. 2006;15(6):802-6.

Jones-Harris AR. Are chiropractors in the uk primary healthcare or primary contact practitioners?: a mixed methods study. Chiropr Osteopat. 2010;18:28.

Stochkendahl MJ, Larsen OK, Nim CG, Axen I, Haraldsson J, Kvammen OC, et al. Can chiropractors contribute to work disability prevention through sickness absence management for musculoskeletal disorders? - a comparative qualitative case study in the Scandinavian context. Chiropr Man Therap. 2018;26:15.

Stochkendahl MJ, Rezai M, Torres P, Sutton D, Tuchin P, Brown R, et al. The chiropractic workforce: a global review. Chiropr Man Therap. 2019;27:36.

Thomas E, Peat G, Harris L, Wilkie R, Croft PR. The prevalence of pain and pain interference in a general population of older adults: cross-sectional findings from the North Staffordshire Osteoarthritis Project (NorStOP). Pain. 2004;110(1-2):361-8.

World Health Organization. WHO global strategy on people-centred and integrated health services. Geneva; 2015.

World Health Organization. World report on ageing and health. Luxemburg: World Health Organization; 2015.
