# Supplementary material for: The chiropractors’ dilemma in caring for older patients with musculoskeletal complaints: Collaborate, integrate, coexist, or separate?
Source: PLoS One. 2024 May 2;19(5):e0302519. doi: 10.1371/journal.pone.0302519 (PMC11065304; doi:10.1371/journal.pone.0302519)
Supplement: Attachment — Submitted filename: Response to reviewers.docx [file pone.0302519.s003.docx]

We appreciate the reviewer’s comments that address limitations in the submitted manuscript. The following letter includes comments on the reviewer’s reports on the submitted manuscript to PLOS ONE.

Review Comments to the Author

Reviewer #1: Thank you for the opportunity to review this paper. I hope my comments and suggestions will be helpful to strengthen the paper.

1. Title: I rarely comment on a title, but the phrase after the colon "collaborate, integrate, coexist, or separate?" was the main reason I agreed to review the paper. Unfortunately I was disappointed because these topics were not really addressed to any significant degree in the paper. Perhaps a title that better reflects the text would be more appropriate?

With the manuscript improvements made based on the reviewers' comments, we hope that the text is now more reflective of the title.

2. sampling/recruitment: In the Design section and again in the methodological considerations section, the authors state that they used purposeful sampling. However, in the recruitment and participants section, the authors write that they recruited a "convenience sample". Purposive sampling is a much stronger choice and if used, more details about how this was implemented (i.e., was was considered in making choices re who to interview) should be included in the recruitment section. Currently it is not at all clear how the participants were selected. Similarly, a justification of the four countries is not provided -- why these countries and not others?

Thank you for this comment. We understand that some clarifications are needed. All participating chiropractors were purposefully selected to participate in the interview. The use of “convenience sample” has been removed, and the correct term is now used throughout the manuscript (page 5). We have also added information regarding the justification for the countries included. For convenience, please see below.

*The countries were selected based on their involvement in the BACE-C and BACE-N (Norway) study.*

The selection of participants has been further explained (page 6 in the manuscript); please see below:

*We aimed to recruit a purposeful sample of twenty chiropractors, five from each of the four countries. These individuals were chosen based on their active involvement in either the BACE-C or BACE-N studies or known to provide care and, on a weekly basis, manage older patients (age 55+) with MSK complaints. Furthermore, all selected chiropractors were members of national chiropractic professional associations in Great Britain, the Netherlands, Norway, and Sweden.*

3. sample size -- no justification was provided for why 24 chiropractors were invited to participate and/or why when only 21 accepted, additional chiropractors were not invited to participate.

We have clarified the flow of participants further in in the manuscript (page 6) as follows:

*In total, we extended invitations for in-depth individual interviews to twenty-four chiropractors via email. However, four declined participation due to various reasons (Fig 1). In addition, the pilot interview (Swedish participant who fulfilled inclusion criteria and also provided a signed consent to participate) was included in the analysis since no changes were made to the interview guide after the pilot interview. Consequently, the sample totalled 21 participants.*

4. Participant demographics (Table 1) -- It would be helpful to know how representative the participants are of the chiropractors in their respective countries. For example, 57% of the participants were women; in some countries the chiropractor profession is male dominated. How des this compare with the sex balance of chiropractors in the four countries from which the participants were sampled? This information would be helpful in assessing the transferability of the findings. I would also have found it helpful to know what proportion of the participants were currently practicing in a private vs public (or both) setting.

We have added information regarding the proportions of participants in private vs. public practice (page 9). For convenience, please refer to the text below:

*All participating chiropractors worked in a private setting; however, two Swedish chiropractors worked in clinics with subcontractor agreements with the public healthcare sector, while all participating Norwegian chiropractors are partially reimbursed for healthcare services provided by the National Insurance Scheme. Some practitioners had prior subcontractor agreements with the public healthcare sector.*

Concerns regarding gender balance have further been discussed under “Methodological considerations”. Please see the text below for convenience:

*Although we aimed for an equal gender distribution, the majority of participants were female (57%), despite chiropractic being a male-dominated profession in the Netherlands (58%), Norway (64.2%) and Sweden (63%). Conversely, in the UK, gender distribution among chiropractors is equal.*

5. analysis: The description of the qualitative analysis procedure was excellent, but I was a bit under-whelmed by the findings as described in the paper. For example:

a. all the participant data appear to have been analyzed as a single data set regardless of the country. Given the different contextual factors in each country (not the least of which that in the Netherlands chiropractors are not regulated), it is not clear why there was no attempt to explore similarities and differences of the barriers and facilitators across participants from different countries.

b. given what is likely a very rich data set, I was a bit disappointed in the depth of the qualitative analysis. As summarized in the conclusion "chiropractors .... emphasized that are well positioned to fill an identified gap regarding care and management of older adults with MSK complaints...... chiropractors saw themselves as an underutilized resource." Despite sections on barriers and facilitators (money and money), there was very little new insight offered. And the subject matter (MSK in the elderly) was not a focus of any of the themes -- what is unique in this patient population vs in others?

Thank you for your valuable feedback. Initially, we anticipated uncovering both similarities and differences during the in-depth individual interviews, given the diverse contextual backgrounds of the countries included in this study. However, to our surprise, we found that there were more similarities than differences despite the varying contexts (such as chiropractors not being licensed in the Netherlands).

Furthermore, due to the substantial amount of information gathered during the interviews (with an average interview duration of over 60 minutes), we faced the challenge of how best to manage this wealth of data to ensure clarity for potential readers. Consequently, we made the decision to divide the material into two parts: one focusing on organisational challenges, integration, and collaboration between health professionals and the other on the treatment and management of older patients. We created this division to make it easier to understand the presentation findings. By splitting the data into categories, we were able to shed more light on the data. However, it's worth noting that other ways of grouping the data may have led to additional insights and conclusions.

Therefore, we have further clarified this in the manuscript (page 8); please see the text below.

*Given the wealth of information collected during the in-depth individual interviews, we opted to split the material into two parts. The first part, focusing on organisational challenges, integration, and collaborations among health professionals, is presented in this paper.*

Additionally, we have also added more information regarding the split in “Methodological considerations” (page 28); please see the text below.

*Nevertheless, the results of this study give insights regarding chiropractors' experiences in the care and management of older patients with MSK complaints with a focus on organisational challenges, integration, and collaboration among health professionals. Given the wealth of information gathered during the in-depth individual interviews, we strategically divided the material to streamline the presentation findings, facilitating a clearer understanding. While our decision to categorise the data undoubtedly helped illuminate the data, it is important to acknowledge that alternative compartmentalisation strategies might have yielded additional insight and conclusion. However, it is noteworthy that analyses regarding the treatment and management of this patient population will be presented in a subsequent paper.*

Analysis regarding the treatment and management of this patient population and what is unique with this patient group (older patients) compared to others is underway, and we eagerly anticipate sharing our findings with the research community and other stakeholders.

6. Discussion The questions of "collaborate, integrate, coexist or separate" in the title where not mentioned at all in the discussion. In fact the vast majority of the discussion was simply an argument about why chiropractors should be used more for supporting elderly patients with MSK concerns and how the data supported this rather than any real discussion of the findings. For example, how are the findings similar or different across countries, regulatory systems, health care systems and funding models? What models seem to be working better/worse? What do the participants think about the options of collaborate, integrate, coexist or separate?

Thank you for your valuable comment. We agree that this topic has not been highlighted enough in the discussion section. Please refer to page 23 in the manuscript or the updated text below.

*Despite variations in the integration and regulation of chiropractic between Great Britain, the Netherlands, Norway, and Sweden, our study revealed several similarities regarding the facilitators and barriers in caring for older patients with MSK complaints. These shared views and experiences underscore a clinical dilemma faced by the participants as they navigate their roles as part of the patient’s healthcare team yet not officially integrated into the wider healthcare systems. Although most participants envisioned a better-integrated future, they also expressed concerns regarding the existing healthcare systems. In their opinion, these healthcare systems were perceived to suffer from challenges such as inadequate funding, staffing shortages, and deficiencies regarding interdisciplinary collaboration and communication. Our findings shed light on significant organisational problems that are perceived to exclude both professionals and patients from accessing nationally funded care, particularly affecting resource-challenged patients or those with limited financial resources. The recognition of these barriers underscores the importance of adaptability and strategic decision-making while simultaneously emphasising a need to address these systemic issues to ensure equitable access to care.*

7. Discussion: The study was focused on caring for older patients with MSK issues, but it wasn't clear what was unique or different or specific with respect to the focus on older patients. This was really not mentioned at all in the results or the discussion. How are the barriers and facilitators any different in this patient group compared to other patient groups?

The manuscript does not discuss barriers and facilitators specific to this patient group compared to others. We made this decision as a consequence of our approach to analysis, with our focus on organisational challenges, integration, and collaboration among health professionals in this manuscript. For more information, please see our response to comment #5.

The team has obviously put a lot of effort into this project and likely has a very rich data set. I hope these comments are helpful in drawing out some additional insights that may make the paper more impactful.

Reviewer #2: Thank you very much for the opportunity to review this interesting work. This a timely well-conducted qualitative study that provides interesting perspectives into a very important topic: conservative care of MSK conditions in older adults.

I would like to commend the authors for conducting such study in this much needed area. The suggestions below are intended to facilitate readers' understanding and richness of this manuscript.

1. Methods, Setting: it may be helpful for the reader to have additional information on how is the payment method for chiropractic care in these countries. Especially as this is discussed by participants and part of the study's results.

We have added additional information about payment methods has been added under the subheading “Setting”. For convenience, please see below:

*In Norway, the majority of chiropractors receive reimbursement for healthcare services from the National Insurance Scheme. In Sweden, certain county councils offer partial reimbursement, and others have subcontractor agreements with chiropractors. However, in Great Britain, chiropractic services are not widely accessible through the National Health Service (NHS). In the Netherlands, chiropractic care may be reimbursed through private healthcare insurance. Consequently, while public and insurance reimbursement schemes do exist, they are not widely available (with the exception of Norway), leaving the majority of patients in these countries to cover their expenses out of pocket.*

2. Methods, recruitment, line 124: was there a criteria for selecting chiropractors who "frequently" manage patients 55+ years? In other words, who did the investigators define "frequently"? For example, chiropractors who saw X or more patients over 55 years old per week?

We acknowledge that the term "frequently" can be ambiguous. Consequently, we have revised the wording to "on a weekly basis" instead. Additionally, we have included information in the results section concerning self-reported estimated percentages of older patients seen per week (pages 6 and 9). For convenience, please see below:

*These individuals were chosen based on their active involvement in either the BACE-C or BACE-N studies or known to provide care and, on a weekly basis, manage older patients (age 55+) with MSK complaints.*

AND

*The participants in this study typically handle a self-estimated patient load ranging from 20% to 80% of older patients with musculoskeletal complaints per week.*

3. Methods, data collection, line 149: suggest including references of the literature that the interview guide was based on. This would support the interview guide content.

A list of references has been added to the interview guide.

4. Methods, data collection, line 150: suggest including additional details on how was the interview guide evaluated. Since only one pilot interview was conducted, detailing the criteria for interview guide evaluation can speak to and support the strength of such guide.

We have added additional information regarding the evaluation of the interview guide on page 7 of the manuscript. For convenience, please see the text below:

*Before data collection started, a pilot interview was performed to evaluate the interview guide. During the pilot interview, close attention was paid to various factors, including the clarity and relevance of questions, the flow and structure of the interview guide, interview length and timing, rapport with the informant, quality of responses, and any challenges encountered. Feedback was then collected from the informant after the interview, covering aspects such as their overall experience, question clarity, coverage of relevant topics, any confusion, redundancy, or irrelevant questions, comfort and interest levels, interview duration, and recommendations for further improvement, resulting in no additional changes*. *As no changes were made to the interview guide, the pilot interview data was added to the data collection with the participant’s signed consent.*

5. Results, table 1: consider including information on participants' practice sector (i.e., public versus private). This is also part of the results and would be helpful to know the number of participants who provided their views for each sector.

Reviewer #1 had a similar request, and additional information regarding public vs. private has been added on pages 5-6 in the manuscript under the subheading “Settings” for further clarification and on page 9 under the subheading “Participants”. For convenience, please refer to the text below:

*In Norway, the majority of chiropractors receive reimbursement for healthcare services from the National Insurance Scheme. In Sweden, certain county councils offer partial reimbursement, and others have subcontractor agreements with chiropractors. However, in Great Britain, chiropractic services are not widely accessible through the National Health Service (NHS). In the Netherlands, chiropractic care may be reimbursed through private healthcare insurance. Consequently, while public and insurance reimbursement schemes do exist, they are not widely available (with the exception of Norway), leaving the majority of patients in these countries to cover their expenses out of pocket.*

*All participating chiropractors worked in a private setting; however, two Swedish chiropractors worked in clinics with subcontractor agreements with the public healthcare sector, while all participating Norwegian chiropractors are partially reimbursed for healthcare services provided by the National Insurance Scheme. Some practitioners had prior subcontractor agreements with the public healthcare sector.*

6. Results, line 392: please clarify what does "immigration background" mean?

Thank you for this comment. Immigration background is defined by the European Commission as a person who has a) migrated into their present country of residence; and/or b) previously had a different nationality from their present country of residence; and/or c) at least one of their parents previously entered their present country of residence as a migrant. We have, therefore, added this definition as a reference in the manuscript (page 18).

 7. Results and discussion regarding participants' perceptions of having more MSK knowledge than the average GP. In the discussion (lines 522-525), authors mention that patients in general health practice are more complex than patients seen by chiropractors (i.e., higher rates of comorbidities, experience longer episodes of LBP, have more sick leave, etc.). But participants have the perception that chiropractors are better suited than other health care professionals to assess and manage these patients. Is this a realistic perception? In other words, if they don't normally see patients as complex as other professions, is this belief that they are better suited actually founded? Or could it be just their perception? Could authors expand on that in the manuscript?

Thank you for bringing this to our attention. We have further expanded on this topic as it also ties in to comment #12. For further information, please refer to page 24 of the manuscript or the below text.

*This perception among participants may arise because chiropractors typically hold a 4- or 5-year master’s degree, whereas physiotherapists typically complete a 3-year bachelor’s degree. Furthermore, there is evidence indicating that GPs may need to improve their understanding and management of LBP to meet international evidence-based standards. Furthermore, the participants' average years in practice surpass 20 years. It is plausible that with such substantial accumulated clinical experience, participants perceive themselves as more competent in managing complex cases within this area compared to other healthcare professionals, and such perceptions contribute to their confidence in addressing complex patient needs. However, these perceptions may very well be unrealistic and further exploration is warranted to assess the extent to which this perceived competence aligns with objective measures of clinical proficiency and patient outcomes.*

8. Discussion, lines 539-547: in results, participants talked about the challenges of free of charge management for patients with low resources and keeping them engaged with active treatment. I.e., the challenges of offering a lower cost to patients who need it, but at the same time motivating their engagement and active participation in their own care. If possible, could authors expand here too and provide a few suggestions from the literature of how this could potentially be approached?

Thank you for your comment. We have expanded the discussion and provided a few suggestions (pages 25). For your convenience, please see the text below.

*While participants generally viewed subsidised care positively, the challenge of balancing affordability with patient motivation to engage in health management was evident. Personalised patient education materials and strong patient-provider communication and trust can enhance health literacy and empower patients to play a more proactive role in their care. Additionally, community-based group exercise initiatives and partnerships with local organisations offer promising avenues enabling older adults to engage in regular, suitable, and health-enhancing physical activity while fostering social interaction. Although the integration of chiropractic into mainstream healthcare and associated subsidies remain patchy or absent, the chiropractic clinician must evaluate the most effective treatment approach while being sensitive to patient preferences and needs to reduce costs and improve patient outcomes and satisfaction.*

9. Discussion, lines 559-561: this was an interesting statement. There are studies reporting positive views of other health care professionals towards chiropractors. Does that mean other professionals' views don't necessarily translate into action and referrals to chiropractors? Or could it be a perception unique to the study participants? Are there any studies regarding referrals to chiropractors from other healthcare professionals that could be used to expand the discussion a bit here too?

Thank you for this comment. We have now further expanded the discussion on this topic. Please refer to pages 26 of the manuscript or the text below for convenience.

*This aligns with prior research findings indicating that primary care physicians generally prefer patients to contact chiropractors independently rather than initiate formal referrals themselves. The hesitancy among healthcare professionals to issue formal referrals may have implications for the efficiency, quality, and safety of patient care within the healthcare system, possibly leading to disruptions in the continuity of care. Because patients mostly pay for chiropractic care out of their own pockets, this may deter both healthcare professionals and patients from pursuing chiropractic treatment options, contributing to the observed reluctance in referrals.*

10. Methodological considerations (or in methods): suggest clearly describing that the interview transcripts were not shared with the participants. This is in the COREQ checklist, but should also be explicitly reported in the manuscript.

The participants did not review the transcripts to ensure interview accuracy; however, alternative validation methods were employed in this study. Notably, researcher triangulation was extensively utilised, as detailed in the Analysis section on pages 8 and 9, lines 193 – 201. This involved multiple authors with diverse backgrounds and experiences reviewing emerging findings, engaging in thorough discussions, and reaching consensus on the interpretations.

11. Conclusion, first sentence: Since this is a qualitative study on chiropractor's perceptions, suggest considering the following re-wording: Chiropractors from Great Britain, Norway, the Netherlands, and Sweden emphasised that they perceive to be well-positioned to fill an identified gap...

This is a mistake on our part, and we agree with the reviewer that it should be reworded. We have now added *“that they perceive to be”* to the sentence.

12. Supplemental material 1, interview guide: The interview guide includes questions about chiropractors education and training to manage this specific population, however this is not reported in the manuscript. Could this potentially provide a rationale for participants' perception of having higher competence in assessing and managing older patients with MSK complaints?

None of the participants had a higher education beyond their chiropractic degree (this information has been added on page 9 in the manuscript). Nevertheless, with a 4- or 5-year master’s degree and an average of over 20 years in practice, we speculate that the length of education (in comparison to other healthcare providers) and the extensive clinical experience contribute to the participants' perceived competence in assessing and managing older patients with musculoskeletal complaints. We have, therefore, expanded the discussion on this topic in the manuscript (page 24). For convenience, please see below:

*This perception among participants may arise because chiropractors typically hold a 4- or 5-year master’s degree, whereas physiotherapists typically complete a 3-year bachelor’s degree. Furthermore, there is evidence indicating that GPs may need to improve their understanding and management of LBP to meet international evidence-based standards. Furthermore, the participants' average years in practice surpass 20 years. It is plausible that with such substantial accumulated clinical experience, participants perceive themselves as more competent in managing complex cases within this area compared to other healthcare professionals, and such perceptions contribute to their confidence in addressing complex patient needs. However, these perceptions may very well be unrealistic and further exploration is warranted to assess the extent to which this perceived competence aligns with objective measures of clinical proficiency and patient outcomes.*
